# A novel cochlear implant assessment tool: Audiometric and speech recognition analysis

**DOI:** 10.1016/j.bjorl.2025.101559

**Published:** 2025-03-05

**Authors:** Fernanda Ferreira Caldas, Byaka Cagnacci Buzo, Bruno Sanches Masiero, Alice Andrade Takeuti, Carolina Costa Cardoso, Fabiane de Castro Vaz, Fayez Bahmad

**Affiliations:** a Universidade de Brasília, Faculdade de Ciências da Saúde, Brasilia, DF, Brazil; b Instituto Brasiliense de Otorrinolaringologia, Brasilia, DF, Brazil; c Cochlear Latin America de São Paulo, São Paulo, SP, Brazil; d Universidade Estadual de Campinas, Faculdade de Engenharia Elétrica e Computação, Campinas, São Paulo, SP, Brazil

**Keywords:** Cochlear implant, Speech perception, Audiometry

## Abstract

**Objective:**

To compare the results of Pure Tone Audiometry (PTA) and speech recognition, of users of Cochlear Implant (CI) between CLABOX with Direct Audio Input (DAI) and the Sound Booth (SB).

**Methods:**

Fifty individuals with CIs, 33 adults and 17 children, were included. Speech recognition tests in noise fixed and adaptive were applied with the Hearing in Noise Test (HINT), Ling test, and PTA, in the SB (in free field) and in CLABOX.

**Results:**

For speech recognition in noise, averages were better in the CLABOX; for PTA, they were higher in the CLABOX; and for Ling test, there was no significant change in the categories between SB and CLABOX. The fixed noise were higher in the CLABOX (88.3%) than in the SB (78.9%), *p*-value < 0.001. In the HINT with adaptive noise, the results were significant (*p*-value = 0.007); the S/N ratio was 2.14 dB in the CLABOX and 3.42 dB in the SB. For the four-tone average, the average thresholds for the CLABOX and the SB were 29.8 dB and 23 dB, respectively (*p-*values < 0.001).

**Conclusion:**

CLABOX was an effective tool to evaluate the PTA and speech recognition tests when compared to the conventional evaluation with a SB in the CI user population.

## Highlights

• Facilitate the cochlear implant programming for both patients and professionals.
• New possibility of evaluation in tests of pure tone and speech recognition.
• Possibility of expanding the use of new evaluation methodologies.
• Improve the viability of clinical care.
• Expand this care model in centers that do not have a sound booth.

## Introduction

Cochlear Implants (CIs) are a reliable option for auditory rehabilitation of patients with severe and/or profound sensorineural hearing loss, but they need to be more widespread in South America.^1^ Worldwide less than 5% of potential cochlear implant candidates have received this treatment, and the vast majority of those who have received it are in high-income countries.^2^

In the postoperative period following CI, the speech processor is programmed to determine the appropriate dynamic range of electrical stimulation for each electrode channel. The dynamic range is the difference between the sound detection threshold (T-level ‒ Threshold level) and loudness ‒ the maximum comfort level (C-level ‒ Comfort level). Psychophysical measurements are digitally stored for each electrode channel, thereby allowing the speech processor to present the encoded sounds at intensity levels that lie between the T and C levels.^3^ To assess and validate this CI programming, it is of great importance that individuals undergo speech recognition and pure tone assessments, which are usually performed in a Sound Booth (SB). Speech recognition tests can be performed in either silence or noise.^4^ The auditory thresholds in the free field indicate the lowest level of sound detection, and speech recognition tests involve linguistic processing at the central level.^5^

Another way to evaluate CIs via speech recognition tests in silence and in noise is with the Direct Audio Input (DAI).^6^ The DAI allows the input signal to bypass the external microphone, thereby eliminating ambient noise oscillations and ambient reverberation at the test site.^7^

Cochlear Corporation launched a new CI assessment tool called the Cochlear Latin America BOX ‒ CLABOX, which uses the DAI connection; therefore, this tool can only be used on individuals with a CI from Cochlear Corporation. The CLABOX enables the assessment of Pure Tone Audiometry (PTA), Ling tests, and speech recognition tests in both silence and noise.

Based on the need for new tools in CI centres to help audiologists evaluate psychoacoustic data and the need to guide programming and validation, this study aims to identify whether the results of pure tone tests and speech recognition tests differ between the CLABOX by DAI and the SB.

## Methods

The study was analysed and approved by the Research Ethics Committee of the Faculty of Health Sciences, all participants and parents/guardians for the children consented to participate in the research. This is an analytical, cross-sectional and prospective study, carried out between 2020 and 2021.

The participants were 50 individuals, including 33 adults (mean of 32.3-years-old) and 17 children (mean of 9.7-years-old). The minimum age was 8-years-old. There were 36 females and 14 males. A total of 38 participants had prelingual hearing loss, and 12 participants had postlingual hearing loss, all with profound bilateral sensorineural hearing loss. Fifteen participants were bilateral CI users, and 35 participants were ere unilateral CI users. All participants had used the CI for at least 6-months, and all CIs were from Cochlear Corporation.

All participants were invited to perform speech recognition tests, the Ling test and PTA. Each test was performed in two conditions, that is, in the SB in the free field as well as in the CLABOX with the DAI connection. Bilateral CI users were evaluated with separate ears in all tests. Tests were performed only in a face-to-face manner.

### Settings for evaluation in the sound booth

A MADSEN brand audiometer, model Itera II, REDUSOM brand SB, serial number 8020 was used. In this evaluation, a specific feature of the speech processor, called SCAN, was deactivated, replicating natural hearing. SCAN captures sound and adapts to its surroundings without manual adjustments. All tests were performed at 0 ° azimuth, one metre from the speaker.

### Settings for evaluation in CLABOX with DAI connection

**Fig. 1:**
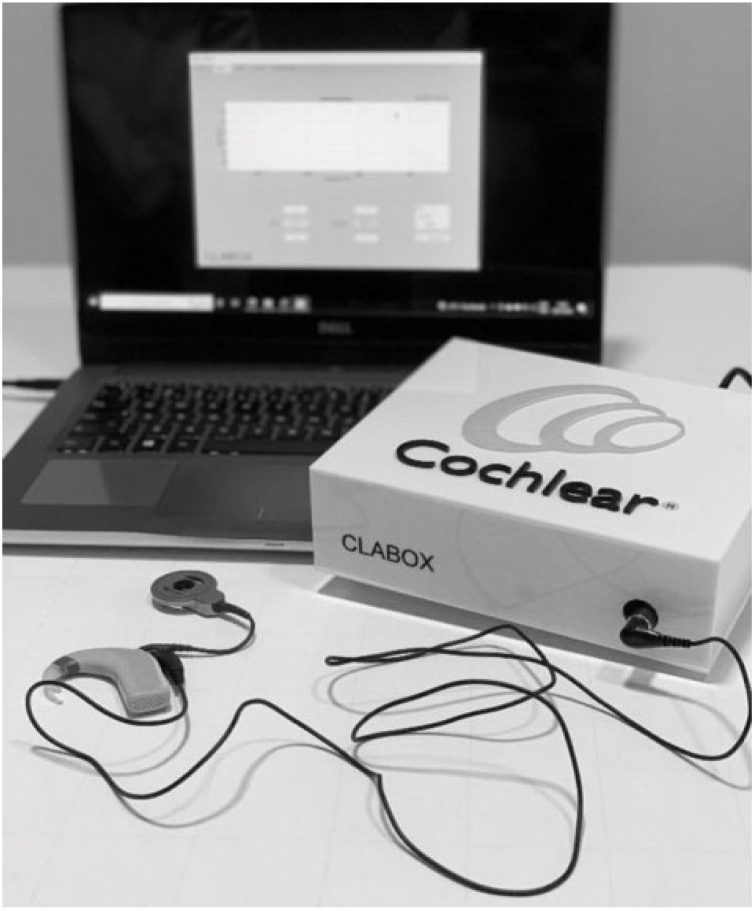
Example of how to connect the CLABOX to the CI via DAI.

To use the CLABOX hardware with the DAI connector (shown in Fig. 1), sound card software (external module) from the Audiobox iOne-Presonus brand was installed on the computer, which enabled the computer to be connected to the CI. The external module has a USB 2.0 audio interface, 24-bit resolution, sampling frequencies of 44.1, 48, 88.2, and 96 Hz, a Class A microphone preamp, +48 V power, a stereo headphone output, a frequency response ranging from 20 Hz to 30 kHz, and an output impedance of 10Ω. The CLABOX was calibrated in accordance with the same standards as the conventional audiometric calibration (ISO 8253) and the manufacturer's information.

The CLABOX presentation software was written in MATLAB with an accompanying Graphical User Interface (GUI), written with MATLAB’s AppDesigner. The GUI has five tabs: a tab for the examiner to input the patient’s data, a tab for the PTA, a tab for the Ling test, a tab for the speech recognition test, and a tab for the examiner to adjust high-level preferences. The software was compiled using the MATLAB compiler as a standalone application. All the required audio files were bundled in the installation package that also included MATLAB runtime.

Initially, the CLABOX was developed to be used only with the CP910 speech processor; in this way, all participants, even CP910 users, had their daily use programs converted to a speech processor used only in search. The speech processor, an external antenna (coil) with magnet, a connector cable between the speech processor and the external antenna, and an audio cable (used only for data collection) were used for connection to the CLABOX. The SCAN feature was also turned off, and the audio cable accessory was in the only option (100% DAI), so the participant heard only the tests directly from the software, and ambient sounds were excluded.^6^, ^8^

### Procedures performed in the sound booth and the CLABOX

#### Pure tone audiometry

To assess pure tone auditory thresholds, frequencies of 250, 500, 1000, 2000, 3000, 4000 and 6000 Hz were investigated in separate ears, with a pulsatile stimulus of 1.5 Hz. The sound intensity was measured in decibels relative to hearing threshold (dB_HL_) and ranged from 0 dB_HL_ to 90 dB_HL_ at intervals of 5 dB. The four-tone average, i.e., the average threshold across the frequencies of 500, 1000, 2000 and 4000 Hz, was also calculated.

#### Ling test

Ling sounds /a/, /i/, /u/, /m/, /s/, /ʃ/^9^ are familiar speech sounds that broadly represent the speech spectrum across the frequency range of approximately 250 to 8000 Hz. This test evaluates approximately the same spectral range as conventional audiometry, and the sounds were presented separately at a fixed level of 65 dB(A) in the recorded condition in separate ears, was presented only in the silent condition. Two auditory skills were evaluated: auditory detection (presence or absence of sound) and auditory recognition (identifying which sound was presented). Participants could speak or point to the corresponding sounds on paper.

#### Speech recognition test

The Brazilian Portuguese version of the Hearing In Noise Test (HINT) was applied.^10^, ^11^, ^12^, ^13^

The software randomly selected a list of sentences, and the examiner manually selected the number of correct words in each sentence presented. A proportion of at least 75% of correct words was expected.

The HINT was applied under two conditions on separate ears: fixed noise, i.e., S/N ratio of +10 dB, 65 dB(A) of speech and 55 dB(A) of noise; and adaptive noise, i.e., noise presented at 55 dB(A) with variables, 4 dB in the initial stage and 2 dB in the final stage.

#### Visual analogue scale

To compare the degree of difficulty or effort between the CLABOX and the SB, after performing each of these tests, the participants used the Visual Analogue Scale (VAS) to indicate the level of difficultly for each test. The scores ranged from 0 to 10. Participants aged between 8 and 13 years were able to use a scale with the different facial expressions for better understanding.

### Statistical analysis

This study used a significance level of 0.05 (5%) and 95% Confidence Intervals. Nonparametric statistical tests were also used (the Mann-Whitney test, the Wilcoxon test, McNemar’s test, Kappa and Spearman’s correlation analysis). To complement the statistical significance, Cohen's *d* (difference) was calculated to determine effect sizes.

Only the participants who obtained results both in the SB and in the CLABOX were analysed. In this way, we obtained paired data, which were examined using the Wilcoxon test.

## Results

**Fig. 2:**
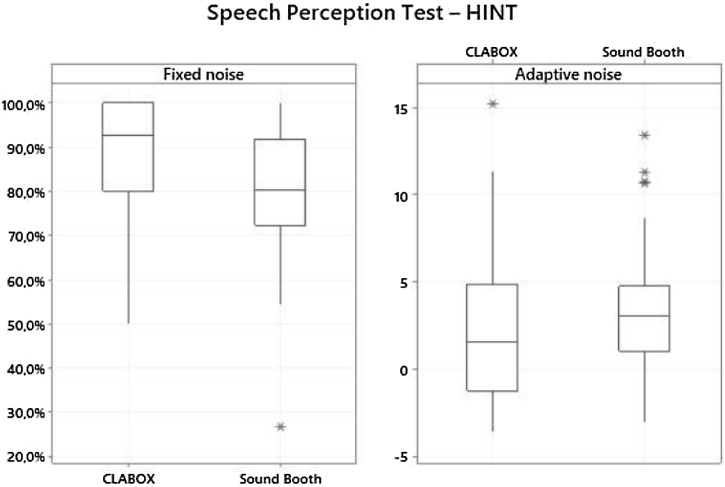
HINT on fixed noise and adaptive noise: comparison of the sound booth and CLABOX systems. The HINT with fixed noise (+10 dB S/N) and the HINT with adaptive noise at 55 dB in the CLABOX and in the sound booth. The box plot represents the 25th and 75th percentiles (box boundaries) and the medians (horizontal line). Outliers are indicated with asterisks.

The results of the speech recognition tests in the SB and the CLABOX are presented in Table 1 and Fig. 2, of the 50 participants evaluated (63 ears), only 40 ears were able to perform the speech recognition test in noise both in CLABOX and SB. There was a difference in the results of the HINT with fixed noise (S/N + 10 dB) and with adaptive noise between the two systems. The average thresholds in the HINT with fixed noise were higher in the CLABOX (88.3%) than in the SB (78.9%), *p-*value < 0.001. In the HINT with adaptive noise, the results were significant (*p*-value = 0.007); the S/N ratio was 2.14 dB in the CLABOX and 3.42 dB in the SB. The boxplot represents the 25th and 75th percentiles (box boundaries) and medians (horizontal line). Outliers are indicated with asterisks. To complement the statistical significance, the effect size (Cohen's *d*) was calculated. In the HINT with fixed noise, there was a medium effect size (0.662); in the HINT with adaptive noise, there was a small effect size (0.331).

**Table 1:** HINT on fixed noise and adaptative noise: comparison of the sound booth and CLABOX systems.

		Average	Median	Standard deviation	Q1	Q3	Nº	CI^d^	*p*-value	Cohen's *d*
HINT Fixed Noise	Sound booth	78.9%	80.3%	15.3%	72.5%	91.8%	40	4.7%	<0.001	0.662
	CLABOX	88.3%	92.5%	13.4%	80.0%	100%	40	4.2%		
HINT Adaptive noise	Sound booth	3.42	3.03	3.60	1.04	4.65	40	1.12	0.007	0.331
	CLABOX	2.14	1.57	4.21	−1.00	4.46	40	1.31		

Q1, Quartile 1; Q3, Quartile 3; Nº, Number; CI, Confidence Interval.

The average threshold for the HINT in fixed noise (88.3%, *p-*value < 0.001) and the HINT with adaptive noise (2.14 dB, *p*-value = 0.007) was higher in the CLABOX than in the sound booth (78.9% and 3.42 dB, respectively). In the HINT with fixed noise, there was a medium effect size (Cohen's *d* = 0.662); in the HINT with adaptive noise, the effect size was small (*d* = 0.331).

**Fig. 3:**
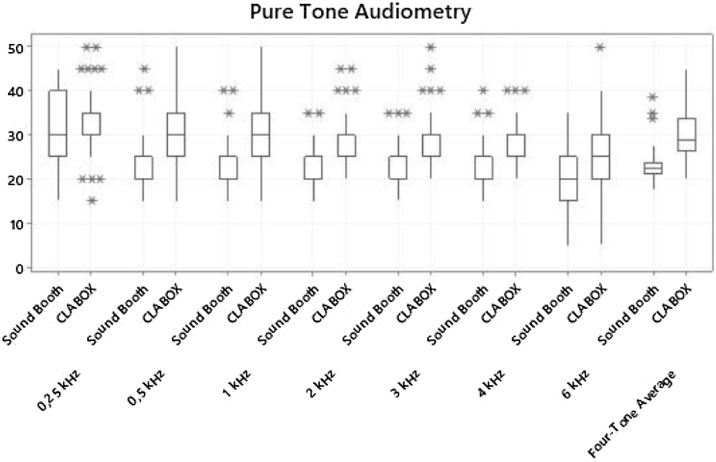
Auditory threshold in pure tone audiometry: comparison of the sound booth and CLABOX systems. Thresholds for the pure tone audiometry assessment in the CLABOX and sound booth. The results were significantly different at all frequencies and for the four-tone average except at 250 Hz. The box plot represents the 25th and 75th percentiles (box boundaries) and medians (horizontal line). Outliers are indicated with asterisks.

Table 2 and Fig. 3 show the results of the pure tone audiometry for each of the evaluated frequencies as well as the four-tone average for the SB and the CLABOX, in this analysis, of the 50 participants (63 ears), all were able to perform the test. There were significant differences between the systems in the thresholds at all frequencies evaluated (*p*-value < 0.001), with the exception of 250 Hz, which had an average threshold of 30.8 dB in the SB and 32.0 dB in the CLABOX (*p*-value = 0.188). At frequencies with significantly different thresholds, the results of the CLABOX were always superior to those of the SB. For example, at 6 kHz, the average thresholds for the CLABOX and the SB were 24.9 dB and 20.3 dB, respectively; additionally, for the four-tone average, the average thresholds for the CLABOX and the SB were 29.8 dB and 23 dB, respectively (*p-*values < 0.001). To confirm the statistical significance, Cohen's *d* was examined. The effect size was small at 250 Hz, the effect size was medium at 6 Hz, and the effect size was very large at the other frequencies and for the four-tone average.

**Table 2:** Auditory threshold in pure tone audiometry: comparison of the sound booth and CLABOX systems.

		Average	Median	Standard deviation	Q1	Q3	Nº	CI	*p*-value	Cohen's *d*
250 Hz	Sound Booth	30.8	30.0	7.9	25.0	37.5	63	1.9	0.188	0.161
	CLABOX	32.0	30.0	7.0	30.0	35.0	63	1.7		
500 Hz	Sound Booth	24.7	25.0	5.9	20.0	25.0	63	1.5	<0.001	1.064
	CLABOX	32.2	30.0	8.2	25.0	35.0	63	2.0		
1 kHz	Sound Booth	24.0	25.0	5.1	20.0	25.0	63	1.3	<0.001	1.115
	CLABOX	30.9	30.0	7.1	25.0	35.0	63	1.8		
2 kHz	Sound Booth	21.7	20.0	4.5	20.0	25.0	63	1.1	<0.001	1.386
	CLABOX	28.9	30.0	5.9	25.0	30.0	63	1.5		
3 kHz	Sound Booth	22.2	20.0	4.8	20.0	25.0	63	1.2	<0.001	1.130
	CLABOX	28.4	25.0	6.1	25.0	30.0	63	1.5		
4 kHz	Sound Booth	21.7	20.0	5.0	20.0	25.0	63	1.2	<0.001	1.109
	CLABOX	27.1	25.0	4.8	25.0	30.0	63	1.2		
6 kHz	Sound Booth	20.3	20.0	5.5	15.0	25.0	63	1.3	<0.001	0.786
	CLABOX	24.9	25.0	6.3	20.0	27.5	63	1.6		
Four-Tone average	Sound Booth	23.0	22.5	3.9	21.3	23.8	63	1.0	<0.001	1.383
	CLABOX	29.8	28.8	5.7	26.3	33.8	63	1.4		

Q1, Quartile 1; Q3, Quartile 3; Nº, Number; CI, Confidence Interval.

In the pure tone audiometry assessment, the average thresholds were higher at all frequencies and for the four-tone average in the CLABOX than in the sound booth (*p*-value < 0.001), except for at 250 Hz (*p*-value = 0.188). The effect size was small at 250 Hz, the effect size was medium at 6 Hz, and the effect sizes were very large at the other frequencies and for the four-tone average.

**Fig. 4:**
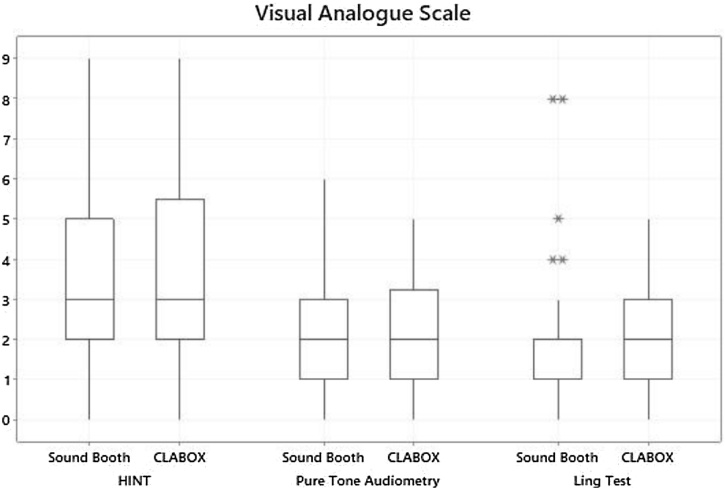
Visual Analogue Scale: comparison of the sound booth and CLABOX systems. VAS results for the HINT, pure tone audiometry and Ling test in the CLABOX and the sound booth. The VAS scores were higher in the CLABOX; however, this difference was not statistically significant. The box plot represents the 25th and 75th percentiles (box boundaries) and medians (horizontal line). Outliers are indicated with asterisks.

The VAS was used to assess the level of difficulty of the speech recognition tests and tone pure in the SB and the CLABOX (shown in Table 3 and Fig. 4). The results indicated that the tests were more difficult in the CLABOX than in the SB; however, the difference was not statistically significant. The effect sizes were very small for the HINT (0.006), PTA (0.162) and Ling test (0.061).

**Table 3:** Visual Analogue Scale: comparison of the sound booth and CLABOX systems.

		Average	Median	Standard deviation	Q1	Q3	Nº	CI	*p-*value	Cohen's *d*
HINT	SB	3.83	3.00	2.13	2.00	5.00	81	0.46	0.773	0.006
	CLABOX	3.84	3.00	2.27	2.00	5.00	81	0.49		
PTA	SB	2.26	2.00	1.61	1.00	3.00	38	0.51	0.372	0.162
	CLABOX	2.50	2.00	1.35	1.25	3.00	38	0.43		
Ling test	SB	1.90	2.00	1.89	1.00	2.00	39	0.59	0.387	0.061
	CLABOX	2.00	2.00	1.50	1.00	3.00	39	0.47		

Q1, Quartile 1; Q3, Quartile 3; Nº, Number; CI, Confidence Interval; SB, Sound Booth.

The average VAS results were higher in the CLABOX; however, the difference was not statistically significant for the HINT test, pure tone audiometry or Ling test. The effect sizes were very small in the HINT (0.006), PTA (0.162) and Ling test (0.061).

In the analysis of the distribution of relative frequencies (percentages) for the Ling test, two statistical techniques were applied: McNemar’s test evaluated whether there was a change in category between the SB and the CLABOX, and the Kappa agreement index assessed whether there was agreement between the responses of the two systems (shown in Table 4). McNemar’s test revealed that for all Ling sounds, there was no significant change in auditory detection and recognition abilities between the two systems. The only exception occurred in the phoneme /i/, where there was a significant change in the categories, with 98.4% of auditory recognition in the SB and 83.9% in the CLABOX. The Kappa value indicated that for the phonemes /u/ and /ʃ/, there was no agreement between the responses of the two systems. The best agreement result occurred in the phoneme /s/, with Kappa (κ = 0.783) (*p*-value < 0.001).

**Table 4:** Ling Test: comparison of Sound Booth and CLABOX systems.

		Auditory detection		Auditory recognition		McNemar	Kappa	
		No	%	No	%		Value	*p*-value
/m/	SB	12	19.4%	50	80.6%	1.000	0.306	0.016
	CLABOX	11	17.7%	51	82.3%			
/u/	SB	6	9.7%	56	90.3%	0.549	0.170	0.169
	CLABOX	9	14.5%	53	85.5%			
/a/	SB	4	6.5%	58	93.5%	0.453	0.307	0.011
	CLABOX	7	11.3%	55	88.7%			
/i/	SB	1	1.6%	61	98.4%	0.004	0.157	0.022
	CLABOX	10	16.1%	52	83.9%			
/ʃ/	SB	2	3.2%	60	96.8%	0.687	−0.045	0.706
	CLABOX	4	6.5%	58	93.5%			
/s/	SB	6	9.7%	56	90.3%	0.500	0.783	<0.001
	CLABOX	4	6.5%	58	93.5%			

Nº, Number; SB, Sound Booth.

For the Ling test, McNemar’s test revealed a statistically significant change in the phoneme /i/, with 98.4% of auditory recognition in the sound booth and 83.9% in the CLABOX. The Kappa value indicates that the average of the phonemes /u/ and /ʃ/ had no significant difference compared to the other phonemes that were significant, while the phoneme /s/ had better agreement between the systems (*p*-value < 0.001).

**Fig. 5:**
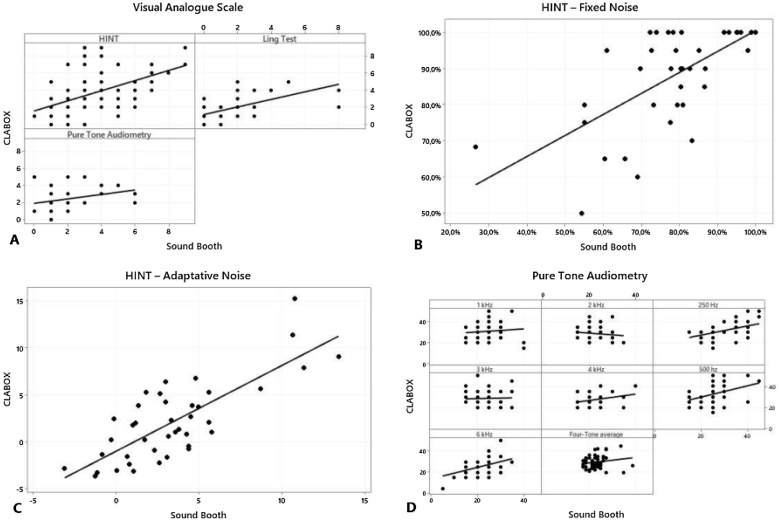
Visual analog scale (A), HINT with fixed noise (B), HINT with adaptive noise (C) and pure tone audiometry (D): correlation between sound booth and CLABOX systems. Correlations were positive and statistically significant (*p-*value < 0.001), mainly in the VAS (A), HINT with fixed noise (B), HINT with adaptive noise (C), and PTA (D). Significant differences were found only at frequencies of 250, 500, 6,000 Hz and for the four-tone average.

To measure the association between the results of the SB and the CLABOX, Spearman's correlation (r)analysis was used. There was a statistically significant positive correlation between the systems, mainly in the VAS and the HINT, *p*-value < 0.001 (shown in Table 5). The strongest correlation was observed in the HINT with adaptive noise (*r* = 0.694) and the HINT with fixed noise (*r* = 0.625), *p*-value < 0.001. There were no significant correlations in the auditory thresholds between the systems at 1,000 Hz to 4,000 Hz; however, there were significant correlations at the lower frequencies, for the four-tone average, and at 6,000 Hz. Fig. 5 presents the data of the individual participants. Comparisons between the CLABOX and the SB are plotted in panels A, B, C and D for the VAS, the HINT with fixed noise, and HINT with adaptive noise and the PTA, respectively. In these figures, it can be seen that when the correlation was positive, the evaluated variable (VAS, HINT and PTA) increased its value proportionally to the other variable that was being correlated, that is, the individual was evaluated with himself. However, if the correlation was negative, the variables were inversely proportional. The most significant correlation was observed in the VAS and HINT and at some frequencies of the PTA.

**Table 5:** VAS, HINT and PTA: correlation between sound booth and CLABOX systems.

		*r*	*p*-value
VAS	Auditory thresholds	0.389	0.016
	HINT	0.581	<0.001
	Ling Test	0.670	<0.001
HINT	Fixed Noise	0.625	<0.001
	Adaptive noise	0.694	<0.001
PTA	250 Hz	0.484	<0.001
	500 Hz	0.379	0.002
	1 kHz	0.196	0.123
	2 kHz	−0.102	0.425
	3 kHz	−0.044	0.735
	4 kHz	0.190	0.136
	6 kHz	0.344	0.006
	Four-Tone average	0.225	0.076

*r*, Spearman’s correlation coefficient, VAS, Visual Analogue Scale, HINT, Hearing in Noise Test, PTA, Pure Tone Audiometry.

There was a significant correlation between the sound booth and the CLABOX system (*p*-value < 0.001) in the VAS, the HINT, at some frequencies on the pure tone audiometry assessment (250 Hz, 500 Hz and 6 Hz) and for the four-tone average.

## Discussion

This study revealed the differences in performance on pure tone assessments and speech tests between the SB and the novel CLABOX tool with DAI connection.

Some studies indicate that remote application with the DAI connection requires high-quality speakers, computers, tablets, or smartphones and that background noise, such as room acoustics in domestic environments, can interfere with speech perception tests.^6^, ^14^

Graaf et al.^4^ and Sevier et al.^8^ de evaluated speech perception among individuals with cochlear implants to compare the feasibility of using DAI in telepractice and in the SB in face-to-face. The results indicated no significant difference between the two modalities; however, there were differences in the results of silent speech tests. In the comparison of auditory performance in the speech-in-noise tests, the participants had significantly better results with the DAI connection than the results with the SB. These data corroborate this study comparing the CLABOX with the SB in the HINTs with fixed and adaptive noise.

In the study by Sevier et al.,^8^ with users of the Cochlear Corporation CI, the scores with the remote DAI connection were 19% in the HINT; 14% in the words of the Consonant-Nucleus-Consonant (CNC) test; and 10% in the phonemes. These scores were worse than those among a person in a SB. Hughes et al.^14^ and de Graaf et al.^6^ also reported significantly lower speech perception test scores in the remote condition with DAI than in the face-to-face condition. The reason for the difference was attributed to higher background noise levels and longer reverberation time at remote locations compared to a SB. In this study, the tests were performed only face-to-face, so this improvement in speech recognition with noise in the DAI system could be attributed to the fact that the connection is direct to the speech processor. Sevier et al.^8^ report that when the input ratio is 100% to the auxiliary port, the DAI connector disables external microphones and presents stimuli directly to the receiver, thereby eliminating reverberation and noise effects background.

In the research of auditory thresholds in a SB, there was a significant difference between the systems in practically all frequencies evaluated, except for 250 Hz. At frequencies with significant differences, the results in the CLABOX were always higher than those in the SB. For example, at 6 kHz, the average thresholds in the CLABOX and the SB were 24.9 dB and 20.3 dB, respectively. The CLABOX produced a small difference in the frequency response compared to the microphone frequency response. This difference is usually not noticed, for example, with music, but it can affect the results of formal audiological tests. It may be that some people with HF are more sensitive to this difference in frequency responses.^6^ For cochlear brand users, there is a difference in the frequency response of the DAI connector versus the speech processor microphones, so this difference may contribute to better results in the DAI condition.^8^

The application of VAS was proposed to quantify the auditory effort that the participants presented between the tests in the two evaluated systems; in this way, the average threshold was higher in the CLABOX, but there was no significant difference compared to the SB. In both systems, there was equivalent effort. Büchner et al.^15^ also used the VAS to assess auditory effort in S/N ratio differences between −10 dB and +15 dB and found a difference in the auditory effort scale for S/N ratios that were rated as difficult (0 dB, −5 dB and −10 dB). Bracker et al.^16^ also reported that the VAS is a useful and sensitive method to quickly assess the listening effort among hearing device users.

It is worth mentioning that despite the differences in the auditory thresholds between the SB and the CLABOX, the participants did not report any difference in the listening effort between these two evaluated systems.

In the Ling sounds, there was no significant change in the categories between the two systems, except for the phoneme /i/, where better recognition was observed in the SB, and the best agreement between the systems occurred in the phoneme /s/. The Ling Test is widely used by parents and therapists to check the detection of six sounds, i.e., /a/, /u/, /i/, /s/, /ʃ/, and /m/, in the frequency range from 250 to 4000 Hz. The detection of all sounds allows auditory access to suprasegmental (speech characteristics in duration, intensity, and frequency) and segmental (vowels and consonant characteristics) features.^9^ The nonsignificant difference found between the systems suggests that the DAI connection can be used in clinical practice with the studied population.

In the correlation analysis between the CLABOX and SB, the plotted values were positive and statistically significant (*p-*value < 0.001), mainly with VAS and HINT. These data are important for clinical practice in the use of the CLABOX, especially with speech perception tests. The data, especially the correlation, between the systems of this study, presents different data from other studies, in which a difference was always found between the SB and the connection with DAI with noise.^4^, ^8^ The study by Chen et al.^7^ showed that the results obtained using direct audio streaming are comparable to those obtained in the SB; however, the authors call attention to the evaluation and consideration of the acoustic characteristics of the environment and equipment.

The results of this study offer new insights into this new tool for collecting and evaluating CIs in pure tone and speech recognition tests, as well as new insights for improvement, such as in places that are difficult to access for evaluation and especially for countries that need to further expand cochlear implant surgeries. It is also worth mentioning that this new tool is easy to handle, is light and small, can be used on a table, and could be used in centres that do not have a SB and free field for evaluation. Some adjustments to the CLABOX software are necessary, mainly in PTA, so that it is a valid assessment tool in clinical routine when a SB and free field are not available in CI centres.

In conclusion, the CLABOX was a valuable tool to evaluate the pure tone and speech recognition tests when compared to the conventional evaluation with a SB in the CI user population.

## CRediT authorship contribution statement

Fernanda Ferreira Caldas conceived and planned the experiments and wrote the manuscript with support from Alice Andrade Takeuti, Carolina Costa Cardoso and Fabiane de Castro Vaz.

Byanka Cagnaci and Bruno Masiero were responsible for developing the CLABOX tool.

Fayez Bahmad Jr. supervised and reviewed the manuscript.

## Publisher’s note

All claims expressed in this article are solely those of the authors and do not necessarily represent those of their affiliated organizations, or those of the publisher, the editors and the reviewers. Any product that may be evaluated in this article, or claim that may be made by its manufacturer, is not guaranteed, or endorsed by the publisher.

## Data availability statement

All data generated or analysed during this study are included in this article. Further enquiries can be directed to the corresponding author.

## Declaration of competing interest

Byanka Cagnacci Buzo is an employee of Cochlear Latin-American. The other authors declare no conflicts of interest to declare.
